# L1, a 3,3′-diindolylmethane-derivative, induced ER stress-mediated apoptosis and suppressed growth through the FLI1/AKT pathway in erythroleukemia HEL cells

**DOI:** 10.3389/fphar.2025.1564199

**Published:** 2025-08-01

**Authors:** Yi Kuang, Yong Jian, Dinghuan Wang, Lihao Bai, Kunlin Yu, Chunlin Wang, Wuling Liu, Sheng Liu, Wan Li, Yaacov Ben-David, Xiao Xiao

**Affiliations:** ^1^State Key Laboratory of Discovery and Utilization of Functional Components in Traditional Chinese Medicine, Guizhou Medical University, Guiyang, Guizhou, China; ^2^ Natural Products Research Center of Guizhou Province, Guiyang, Guizhou, China; ^3^Institute of Materia Medica, Chinese Academy of Medical Science and Peking Union Medical College, Beijing, China

**Keywords:** erythroleukemia, 3,3′-diindoylmethane derivatives, endoplasmic reticulum stress-mediated apoptosis, FLI1, Hsp70

## Abstract

**Introduction:**

3,3′-Diindolylmethane (DIM) is a major phytochemical product derived from ingestion of cruciferous vegetables. As an effective cancer chemopreventive agent, DIM has been used in preclinical and clinical trials. Recently, our group synthesized and modified a novel DIM derivative, L1, and demonstrated its significant antileukemic activities.

**Methods:**

MTT assay was used to confirm the inhibition rates and IC_50_ value of L1 in erythroleukemia HEL cells. Flow cytometry analysis was used to reveal cell cycle arrest and apoptosis. RNAseq data with KEGG pathway enrichment analysis was performed to predict the anticancer mechanism of L1. RT-qPCR and Western blotting were carried out to verify the mechanism in the ER stress-mediated apoptosis and FLI1/AKT pathway. FLI1 knockdown in HEL cells was performed to confirm the mechanism of L1 in the FLI1/AKT pathway. AutoDocking analysis and PPI analysis via the STRING database were used to discover the potential target of L1. HSPA1A knockdown and treatment with HSP70 inhibitor were used to further evaluate the L1 target.

**Results:**

L1 significantly inhibited the growth of erythroleukemia HEL cells, with an IC_50_ value of 1.15 ± 0.03 µM L1 induced G2/M cell cycle arrest and cell apoptosis. RNA sequencing analysis revealed that differentially expressed genes (DEGs) mainly enriched in protein processing of endoplasmic reticulum (ER). L1 increased the protein expression level of GRP78 (BIP) and the RNA transcription of XBP1 and DDIT3 to induce ER stress-mediated apoptosis. Meanwhile, PPI analysis suggested that HSP70 (HSPA1A and HSPA1B) is a pivotal gene that may be involved in the ER stress. AutoDocking analysis also revealed that L1 may bind to the HSP70 protein (HSPA1A and HSPA1B). The apoptosis rate was reduced by cotreatment of L1 and the Hsp70 inhibitor VER155008. Moreover,the inhibition rate was decreased in the HSPA1A knockdown HEL cells, suggesting that L1-induced apoptosis was related to HSP70 activity. Moreover, FLI1 is a crucial target for mediating cell differentiation, apoptosis, inflammation and displays abnormal expression in HEL cells. Here, we showed that the protein expression levels of FLI1 and AKT/p-AKT decreased with L1 treatment and that the RNA expressions of their downstream genes GATA1, TFRC, GYPA, CDKN1A and CDKN1B were also regulated by L1.

**Conclusion:**

This study revealed that the DIM-derivative molecule, L1, induced ER stress-mediated apoptosis and suppressed cell growth by inhibiting the FLI1/AKT pathway in erythroleukemia HEL cells.

## 1 Introduction

Acute myeloid leukemia (AML) is a malignant clonal blood system disease accompanied by abnormal proliferation and impaired differentiation of hematopoietic progenitor cells (Thol and Ganser, 2020; Kantarjian et al., 2024). Acute erythroid leukemia (AEL) is a subtype of acute myeloid leukemia (AML) that accounts for less than 5% of all AML. AEL, which progresses faster and has a worse prognosis than other AML, is characterized mainly by malignant hyperplasia of proto-erythrocytes and erythrocytes to dominate in the bone marrow (≥50%) (Weinberg and Arber, 2021). While daunorubicin and cytarabine achieved partial clinical remission according to the standard chemotherapy regimen, Stem cell transplantation (SCT) is considered a potential treatment strategy for AEL (Fernandes et al., 2024). However, despite great advances in the treatment of AEL patients over the past few decades, there is no significant improvement in the therapeutic outcomes of patients. Common problems in the clinic include primary induction failure (PIF), relapse, and toxicity of chemotherapy drugs (Liao et al., 2021). Many hemotherapy drugs that cause adverse reactions, such as gastrointestinal reactions and bone marrow suppression, not only damage normal tissues and cells but also lead to decreased immunity. Therefore, there is an urgent need to find safer and more effective drugs for AEL treatment.

Natural products are important sources for the development of new anticancer drugs. 3,3 Indole-3-Carbinol (I3C) is released as a hydrolysis product of glucoerucin, which is derived from Brussels sprouts and other cruciferous vegetables, such as broccoli and cauliflower (Williams, 2021). 3,3′-Diindolylmethane (DIM) is the major acid condensation product, produced by I3C *in vivo*. As shown in several studies, DIM has been developed as a potential chemotherapeutic compound for cancer prevention and therapy. In terms of mechanism, DIM can bind to aryl hydrocarbon receptor (AHR), estrogen receptor and androgen receptor (AR) as agonists or antagonists to inhibit proliferation and induce apoptosis in several cancer cell lines (Williams, 2021). Nevertheless, the undesirable solubility and poor bioavailability of DIM *in vivo* limit its clinical application. Moreover, although DIM has been reported mainly in human clinical trials for the treatment of breast and prostate cancer, its efficacy in acute myeloid leukemia has seldom been explored.

In a previous study, our research group optimized this compound through structural modification, and a novel 3,3′-diindolemethane (DIM) derivative (L1) was obtained during drug screening for anti-AML activities. This study evaluates the anti-erythroleukemia activities of the DIM derivative L1 *in vitro* and revealed that L1 induced ER stress-mediated apoptosis and mediated the FLI1/AKT pathway in HEL cells. These data demonstrated that the DIM derivative L1 was a potential candidate for erythroleukemia therapies.

## 2 Methods

### 2.1 Cell lines and cell culture

Verified *mycoplasma* negative tested human cell lines originated from ATCC. Erythroleukemia (HEL), chronic myelogenous leukemia (K562) and T lymphoblastic leukemia cell (CEM-C1) were cultured in RPMI, and the breast cancer (MDA-MB-231, MCF7), and normal liver cell (HL-7702) were cultured in DMEM medium (high glucose), with supplemented with 5%–10% fetal bovine serum (HyClone, GE Healthcare, Australia). These cells were maintained in a humidified incubator containing 5% CO_2_ at 37°C. After growing to approximately 70%–90% confluence cells were treated with compounds. During cell culture, real-time monitoring of cells on the gene expression and phenotypic changes, regular screening and identification were carried out at regular period to avoid genetic drift.

### 2.2 Cytotoxicity assay

Triplicates of cancer cell lines (8 × 10^3^/well) were plated 96-wells plates and incubated with various concentrations of compound for 72 h. The dose-dependent effect of compound on cell viability was assessed by adding diphenyltetrazolium bromide (MTT, 5 mg/mL) reagents (Solarbio, Beijing, China) to each well for 4 h. The formazan crystals were dissolved in designated solution (100 g/L SDS, 1 mL/L HCL, 50 mL/L 2-Methyl-1-propanol). Optical density (OD) was measured with the Synergy2 Reader (BioTek, United States). The IC_50_ values was determined accordingly (Dinghuan et al., 2024). The cellular morphology of the HEL cells was observed in Nikon microscope (Leica, Germany).

### 2.3 Cell cycle and apoptosis

Cell cycle and apoptosis analysis performed according to published methods (Long et al., 2021). In brief, cells were incubated with L1 or 0.5% DMSO for 24 h and then washed twice by cold PBS. For apoptosis detection, cells were stained by Annexin V-FITC and Propidium Iodide (PI) apoptosis detection Kit (BD Biosciences, United States) following the kit guidelines, and analyzed by NovoCyte flow cytometer (NovoCyte, Aglient, United States). For cell cycle analysis, cells were fixed by iced 70% ethanol at 4°C overnight, washed once with cold PBS, stained in PI (Solarbio, Beijing, China) for 30 min in the dark at RT (25°C). The cellular DNA content was analyzed by NovoCyte flow cytometer (NovoCyte, Aglient, United States).

### 2.4 Western blotting and inhibitory compound

Western blotting was done using protocol, as previously described (Dinghuan et al., 2024). The antibodies used are as follows: PARP (#9542), Casepase3 (#9662) were purchased from Cell Signaling Technology (United States), HSP70 (#382481); CHOP (#381679), BIP (#200310-4F11), AKT (#342529), p-AKT (#341790) were purchased from ZEN-BIO (China); the goat anti-mouse (5470 S) and goat anti-rabbit (5151 S) HRP conjugated antibodies were purchased from Cell Signaling Technology (United States); the GAPDH (#AB-P-R001) antibody was obtained from Hangzhou Goodhere Biotechnology. Antibody dilution conducted according to the manufacturer’s instructions. The Oddessy system (Li-Cor Biosciences, Lincoln, United States) used for protein detection. VER155008(HY-10941) was obtained from MedChemExpress (New Jersey, United States) and used in some experiments.

### 2.5 RNA preparation and RT-qPCR

Total RNA was isolated from cells with TRIzol reagent (Life Technologies, Thermo Fisher Scientific, United States) and then cDNA was synthesized using PrimeScript RT Reagent Kit (Cat# RR047A, Takara, Beijing, China) according to manufacturer’s protocol. RT-qPCR performed by FastStart University SYBR Green Master kit (Cat# 04913914001, Roche, Germany) and the Step One Plus Real-time PCR system (Applied Biosystems, Thermo Fisher Scientific, US). The expression of the test genes was calculated as relative values to the expression of GAPDH using the 2^−ΔΔCt^ method. Three biological replicates were conducted for all RT-qPCR experiments, each in triplicate (*n* = 3). Primer sequences were shown in Table 1 (Some primer sequences were acquired from PrimerBank, Harvard University).

**TABLE 1:** RT-qPCR gene primers sequence.

Gene	Forward	Reverse
XBP1	CCC​TCC​AGA​ACA​TCT​CCC​CAT	CCC​TCC​AGA​ACA​TCT​CCC​CAT
DDIT3	GGA​AAC​AGA​GTG​GTC​ATT​CCC	CTG​CTT​GAG​CCG​TTC​ATT​CTC
FLI1	CAG​CCC​CAC​AAG​ATC​AAC​CC	CAC​CGG​AGA​CTC​CCT​GGA​T
miR145	TCC​CTA​AGG​ACC​CTT​TTG​ACC	AGT​CTC​AGG​GTC​CGA​GGT​ATT​C
U6	CTCGCTTCGGCAGCACA	AAC​GCT​TCA​CGA​ATT​TGC​GT
GATA1	CTG​TCC​CCA​ATA​GTG​CTT​ATG​G	GAA​TAG​GCT​GCT​GAA​TTG​AGG​G
TFRC	ACC​ATT​GTC​ATA​TAC​CCG​GTT​CA	CAA​TAG​CCC​AAG​TAG​CCA​ATC​AT
GYPA	ACA​ACT​TGC​CCA​TCA​TTT​CTC​TG	TCA​GTC​GGC​GAA​TAC​CGT​AAG
HSPA1A	GCC​GAG​AAG​GAC​GAG​TTT​GA	GAA​GCT​CCA​AAA​CAA​AAA​CAG​CA
HSPA1B	GGT​GGA​TTA​GGG​GCC​TTT​GT	ACA​GCA​GCA​AAG​TCC​TTG​AGT
GAPDH	CTG​GGC​TAC​ACT​GAG​CAC​C	AAG​TGG​TCG​TTG​AGG​GCA​ATG
CDKN1A	TGT​CCG​TCA​GAA​CCC​ATG​C	AAA​GTC​GAA​GTT​CCA​TCG​CTC
CDKN1B	TGC​AGG​TCG​CTT​CCT​TAT​TCC	TGC​AGG​TCG​CTT​CCT​TAT​TCC
BIP	CTG​GGT​ACA​TTT​GAT​CTG​ACT​GG	GCA​TCC​TGG​TGG​CTT​TCC​AGC​CAT​TC

### 2.6 RNAseq data analysis

RNAseq was performed using HEL cells treated with L1 (1 µM) or 0.5% DMSO for 24 h and sequencing was performed on Illumina NovaSeq 6000 with PE150 (read length) by Shanghai Origingene Institute (China). After obtaining clean reads, HISAT2 (v2.1.0) was used to align clean reads to the reference genome sequence. Differentially expressed genes (DEG) were identified from RNAseq data and used for the KEGG pathway analysis. Heatmaps were used to display the list of genes associated with protein processing in endoplasmic reticulum signaling. The interaction of HSPA1A/HSPA1B with other proteins were analyzed using STRING database (https://cn.string-db.org) (Szklarczyk et al., 2022). The setting interaction was graphed using the default medium confident setting (a minimum required interaction of 0.4).

### 2.7 Computer docking

The protein crystallographic structure of receptors,HSP70/HSPA1A (PDB:3JXU) and HSPA1B(PDB:7F4Z), were derived from www.rcsb.org. Auto Dock tools 1.5.7 (software available at https://autodocksuite.scripps.edu/adt/, California, United States) (Morris et al., 2009) was used to compute the molecular docking simulations according to the standard protocol in the software documentation. Furthermore, the interacting sites were analyzed using PyMOL2.1 analysis (software available at https://pymol.org/, New York, United States) (Schrödinger and DeLano, 2020).

### 2.8 ShRNA expression

The construction of shRNA lentiviruses was generated, as previously described (Dinghuan et al., 2024). Briefly, shHSPA1A and scrambled control vectors were constructed by inserting the shHSPA1A and scrambled DNAs into the restriction enzyme sites within the Plent-GFP expression vector that was obtained from Vigene Bioscience (Rockville, MD, United States). To produce life lentivirus particles, the shHSPA1A expression plasmid (10 µg), packing plasmids psPAX2 (5 µg) and pMD2. G (10 µg) (Addgene plasmid #12259 and #12260) were co-transfected into HEK293T cells using Lipofectamine 2000 (Thermo Fisher Scientific, US). Forty-eight hours post transfection, the supernatants were harvested, the viruses were centrifuged at 1,000 *g* for 10 min, filtered through 0.45 µm filters, and then used for infection freshly or stored at −80°C. For leukemic cells infections, the HEL cells were cultured in the presence of fresh virus-containing supernatant. After 24 h infection, the medium was changed and positive cells were selected after with medium containing puromycin (5 μg/mL) (Solarbio, Beijing, China). The sequences of shHSPA1A lentiviruses are as follow:

**Table:** 

shRNA	Forward
shHSPA1A-1	GGT​GCT​GAC​CAA​GAT​GAA​GGA​TTC​AAG​AGA​TCC​TTC​ATC​TTG​GTC​AGC​ACC​TTT​TTT
shHSPA1A-2	AGC​GCA​ACG​TGC​TCA​TCT​TTG​TTC​AAG​AGA​CAA​AGA​TGA​GCA​CGT​TGC​GCT​TTT​TTT
shHSPA1A-3	CCA​TGA​CGA​AAG​ACA​ACA​ATC​TTC​AAG​AGA​GAT​TGT​TGT​CTT​TCG​TCA​TGG​TTT​TTT

ShFLI1 HEL cells were acquired as previous description (Song et al., 2018).

**Table:** 

shRNA	Forward
shFLI1-1	GCG​TCA​TGT​TCT​GGT​TTG​AGA​TTT​CAA​GAG​AAT​CTC​AAA​CCA​GAA​CAT​GAC​GTT​TTT​T
shFLI1-2	GCC​CTT​CTG​ACA​TCT​CCT​ACA​TTT​CAA​GAG​AAT​GTA​GGA​GAT​GTC​AGA​AGG​GTT​TTT​T
shFLI1-3	GCC​CAT​GAA​CTA​CAA​CAG​CTA​TTT​CAA​GAG​AAT​AGC​TGT​TGT​AGT​TCA​TGG​GTT​TTT​T

### 2.9 Statistical analysis

A statistical analysis was performed using a two tailed Student’s t-test or a one-way ANOVA with Tukey’s *Post Hoc* Test with significance considered at *p <* 0.05 (*), *p <* 0.01 (* *), and by analysis of variance using GraphPad Prism9 software (software available at https://www.graphpad.com/features, Boston, United States). The 95% Confidence Intervals (CI) of the sample mean were constructed. For the independent samples T-test, Cohen’s *d* is determined by calculating the mean difference between two group.

## 3 Results

### 3.1 L1 inhibits leukemia cell growth and proliferation

**FIGURE 1:**
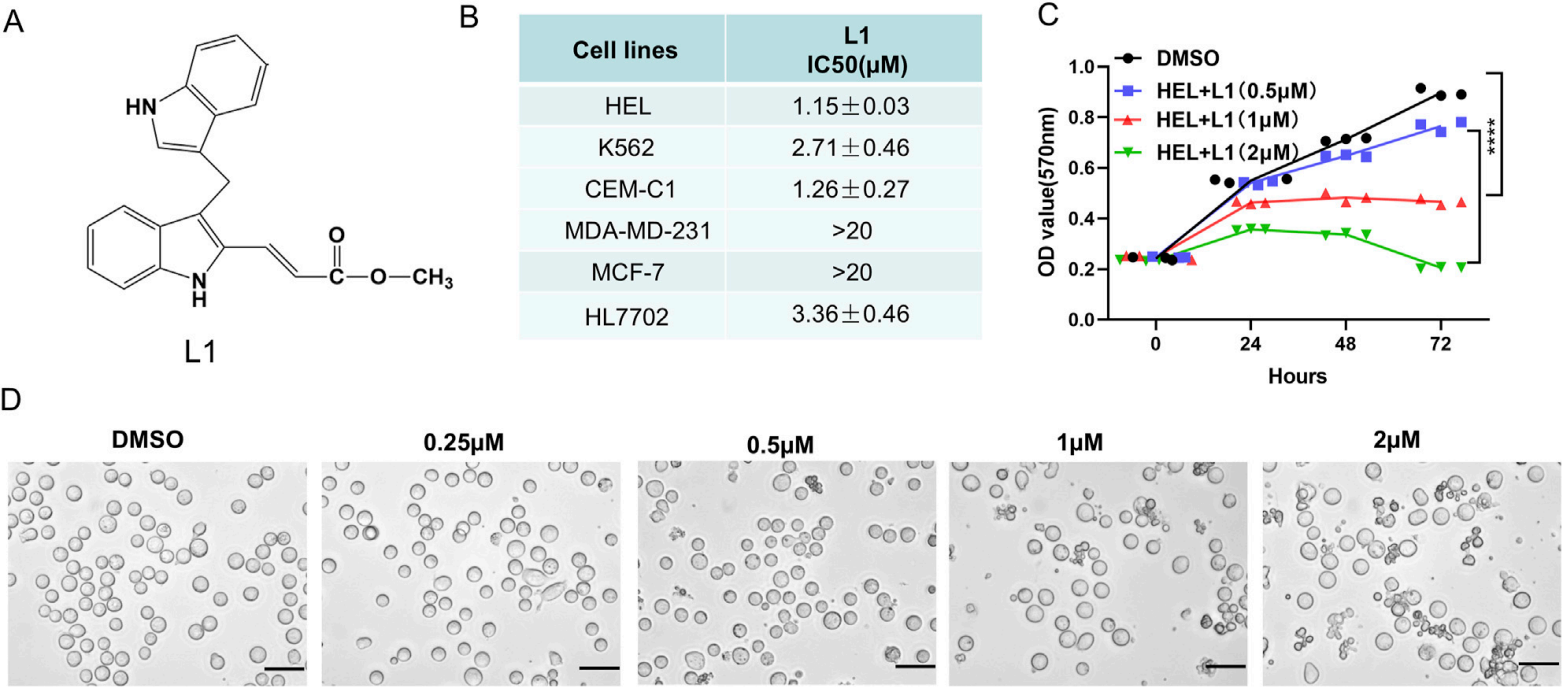
L1 inhibits the cell growth and proliferation in leukemia cell lines. **(A)** The chemical structure of L1. **(B)** The IC50 values of L1 on leukemia cell lines HEL, K562,CEM-C1 at 72 h were detected by MTT assay. The cells were treated with various concentrations of L1 (0.3125, 0.625, 1.25, 2.5, 5 μM) at 72 h. **(C)** The optical density changes of viable HEL cells after L1 treated were analyzed by MTT assay in concentration-dependent manner. The cells were treated with various concentrations of L1 (0.5, 1, 2 μM) at 24 h, 48 h and 72 h. **(D)** The morphology images in the HEL cells were observed by microscopy after L1 treatment (0.25, 0.5, 1, 2 μM) for 24 h. Scale bar: 40 μm *p < 0.05, **p < 0.01, ***p < 0.001, versus control, n = 3.

Our group synthesized and modified a new DIM derivative, L1 (Figure 1A). After treatment for 72 h, L1 significantly and selectively inhibited the growth of the leukemia cell lines HEL, K562, and CEM-C1, with IC_50_ values of 1.15 ± 0.03 µM, 2.71 ± 0.46 µM, and 1.26 ± 0.27 µM, respectively. However, the IC_50_ values were greater than 20 µM in the breast cancer cell lines MBA-MD-231 and MCF7, while the IC_50_ values in the normal cell lines HL7702 was 3.36 ± 0.46 µM, respectively (Figure 1B). L1 impaired the viability of erythroleukemia HEL cells in a time- and concentration-dependent manner (Figure 1C). As the concentration of L1 increased, HEL cell morphology became more fragmented (Figure 1D). These data revealed that L1, a DIM derivative, has antileukemic activity.

### 3.2 L1 induces G2/M cell cycle arrest and cell apoptosis in HEL cells

**FIGURE 2:**
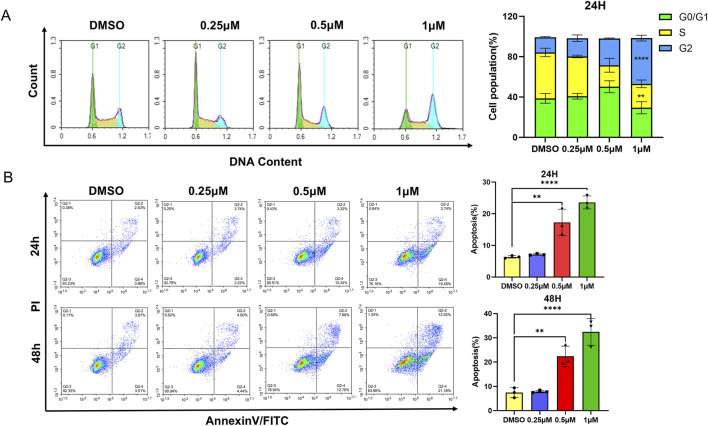
L1 induces cell cycle arrest and apoptosis. **(A)** The HEL cells were incubated with different concentrations of L1 (0.25, 0.5, 1 μM) for 24 h, stained with propidium iodide, and analyzed by flow cytometry. **(B)** The HEL cells were treated with various concentrations of L1 (0.25, 0.5, 1 μM) for 24 h and 48 h, and stained with annexin V-FITC and PI. The flow cytometry was used to detect the cell apoptosis. *p < 0.05, **p < 0.01, ***p < 0.001, versus control, n = 3.

The cell cycle and apoptosis were analyzed via flow cytometry in the HEL cell line. L1 induces cell cycle arrest at the G2/M phase after L1 treated for 24 h (Figure 2A). The rates of the G2/M phase were 18.00% ± 3.25%, 26.58% ± 0.43% and 45.40% ± 2.84% at L1 concentrations of 0.25 µM, 0.5 µM, and 1 μM, respectively. L1 induced cell apoptosis at concentrations of 0.5 µM and 1 µM for 24 h and 48 h. The apoptosis rates were 17.27% ± 4.2% and 22.39% ± 4.2% at 0.5 µM, and 23.59% ± 2.06% and 32.45% ± 5.54% at 1 µM for 24 h and 48 h, respectively (Figure 2B). These data suggested that L1 inhibits leukemia through induction of cell cycle arrest and apoptosis.

### 3.3 L1 induces endoplasmic reticulum (ER) stress-mediated apoptosis

**FIGURE 3:**
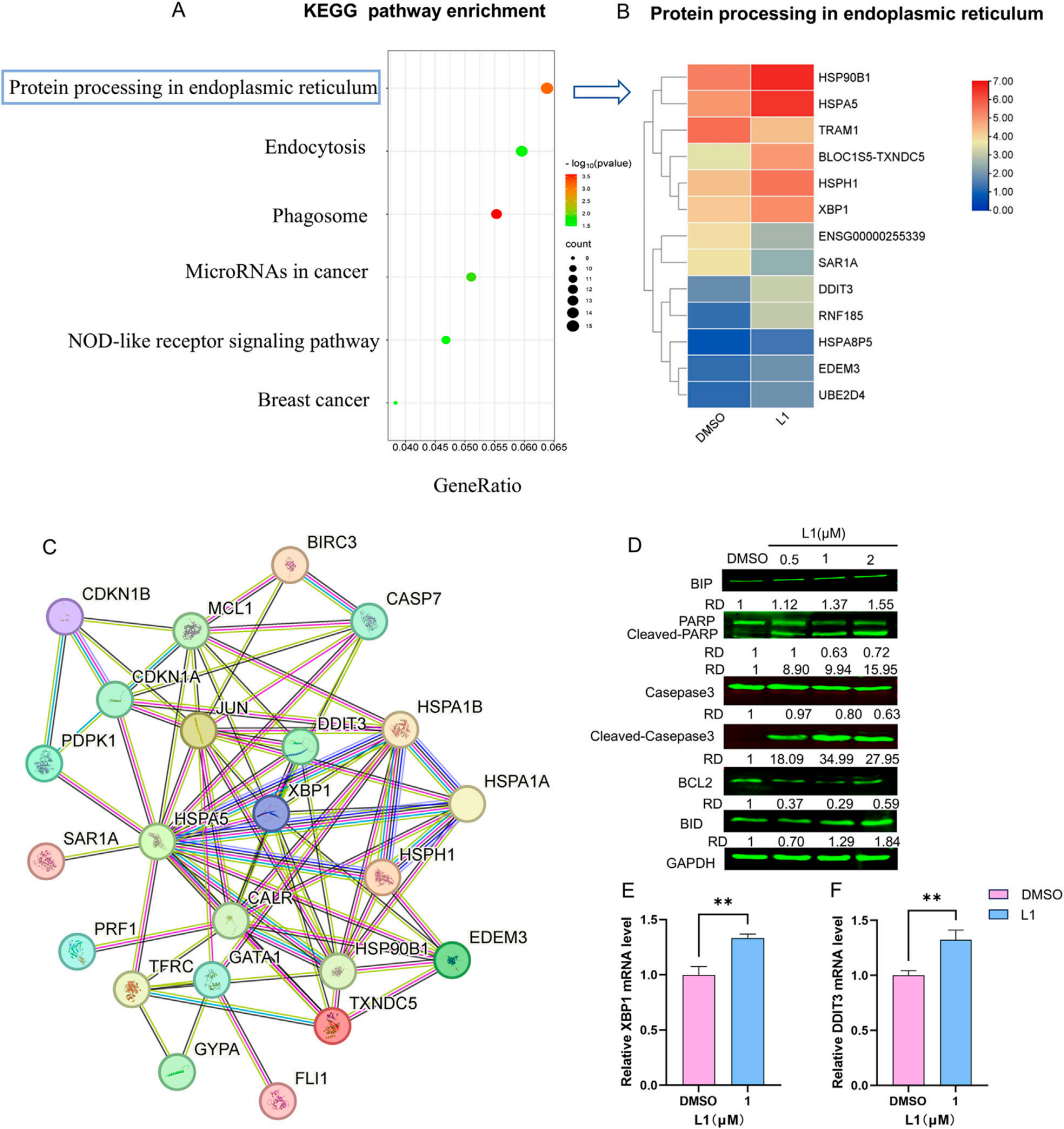
L1 induces ER stress-mediated apoptosis. **(A)** The KEGG pathway enrichment analysis for the DEGs. **(B)** The DEGs heatmap of protein processing in endoplasmic reticulum. **(C)** The PPI interaction network of genes in pivotal pathway. The nodes denoted the core genes and the edges displayed the interactions between these genes. **(D)** The expression levels of BIP, PARP, Cleaved-PARP, Casepase3, Cleaved-casepase3, Bcl-2 and BID were detected by Western blot assay. The HEL cells were treated with vehicle control, L1 (0.25, 0.5, 1 μM) for 24 h. GAPDH expression was used as reference standard for quantification. The relative expression (RD) was used to quantified the protein expression. **(E, F)** The expression levels of XBP1 and DDIT3 were detected by RT-qPCR. The HEL cells were treated with vehicle control (0.1%DMSO) or L1 (1 μM) for 24 h *p < 0.05, **p < 0.01, ***p < 0.001, versus control, n = 3.

To confirm the mechanism of L1 in HEL cells, the KEGG pathway of differentially expressed genes (DEGs) enriched in protein processing in the endoplasmic reticulum was identified via transcriptome sequencing analysis (Figure 3A). After L1 treatment, the expression levels of the heat shock protein genes HSP90B1, HSPA5, and HSPH1 were upregulated, and the expression levels of the XBP1 and DDIT3 genes related to the endoplasmic reticulum pathway were also upregulated, as shown in the heatmap (Figure 3B). Protein-protein interaction analysis via STRING analysis revealed that the greatest number of edges for HSPA1A/HSPA1B were connected to other DEGs, which may be pivotal nodes regulating the endoplasmic reticulum stress-related apoptosis pathway (Figure 3C). Immunology analysis revealed that the expression of the endoplasmic reticulum-related protein BIP (Grp78/HSPA5) was upregulated. The expression levels of the apoptosis-related proteins cleaved-PARP, cleaved-caspase three and Bid were also increased, whereas the expression of the antiapoptotic protein BCL-2 was decreased (Figure 3D). Moreover, the gene expression levels of XBP1 and DDIT3 were increased by L1, as detected by RT-qPCR (Figures 3E,F). These data revealed that L1 induced apoptosis by modulating the ER stress.

### 3.4 L1 modulated the miR145//FLI1/AKT signaling pathway to suppress proliferation

**FIGURE 4:**
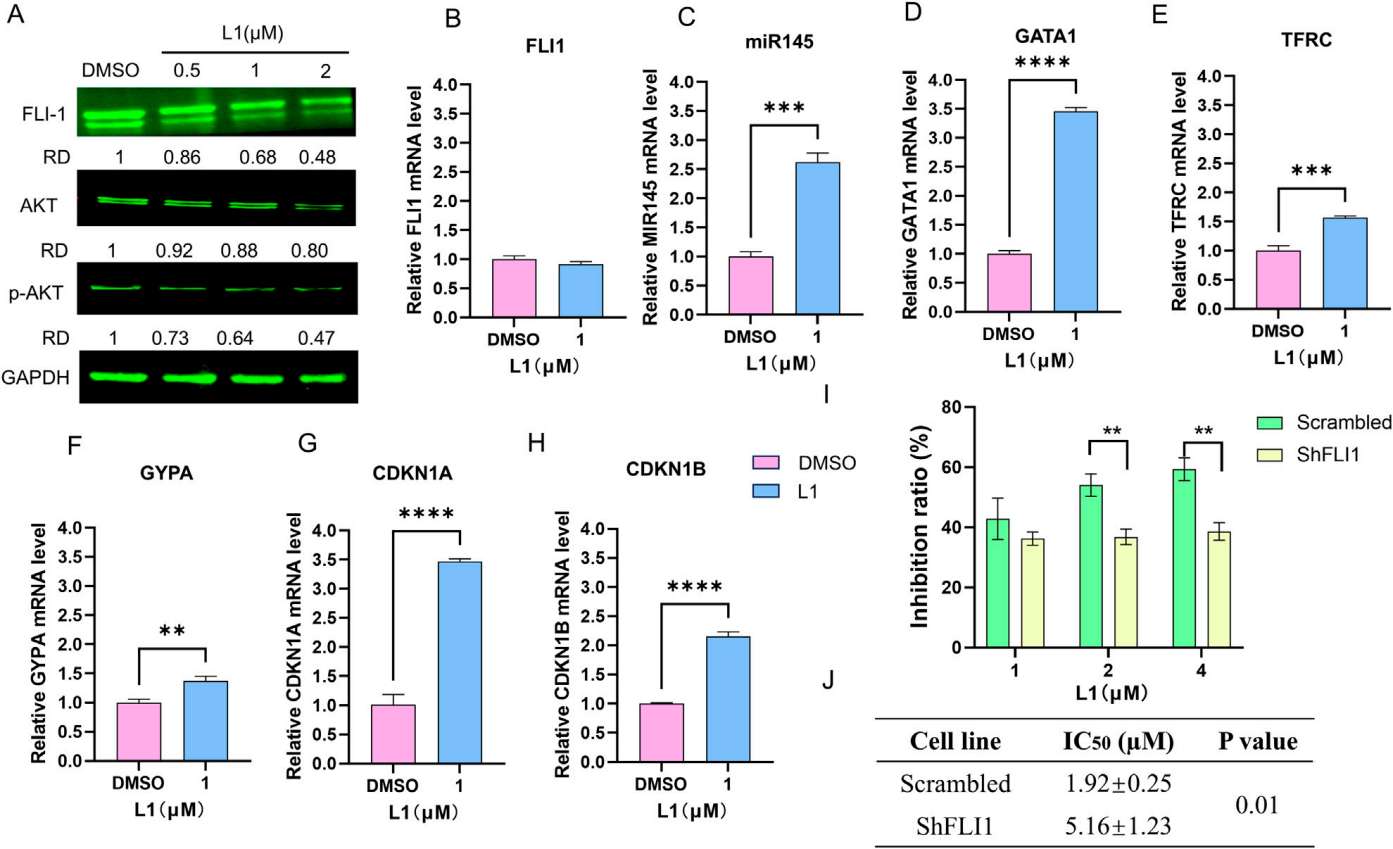
L1 modulated miR145/Fli-1/AKT signaling pathway. **(A)** The expression levels of FLI1, AKT and p-AKT were detected by Western blot assay. The HEL cells were treated with vehicle control, L1 (0.5, 1, 2 µM) for 24 h. GAPDH expression was used as reference standard for quantification. The relative expression (RD) was used to quantified the protein expression. **(B–H)** The expression levels of FLI1, miR145, GATA1, TFRC, GYPA, CDKN1A and CDKN1B were detected by RT-qPCR. The HEL cells were treated with vehicle control (0.1%DMSO) or L1 (1 µM) for 24 h. **(I)** The inhibition rates of L1 in the HEL cells with shFLI1 knockdown were detected by MTT assay. The HEL cells were treated with vehicle control (0.1%DMSO) or L1 (1, 2, 4 µM) for 72 h. **(J)** The IC_50_ values of L1 at 72 h in leukemia cell lines HEL with shFLI1 knockdown and scrambled vector were detected by MTT assay. The cells were treated with various concentrations of L1 (0.5, 1, 2, 4 μM) at 72 h *p < 0.05, **p < 0.01, ***p < 0.001, versus control, *n* = 3.

FLI1, an ETS transcription factor, is involved in mediating hematopoietic stem/progenitor cell differentiation, death and inflammation and is aberrantly expressed in HEL cells (Ben-David et al., 1990; Li et al., 2015). The protein expression level of Fli-1 was downregulated (Figure 4A), while its transcription remained unchanged in the L1-treated HEL cells (Figure 4B). The microRNA, miR145, has been previously shown to negatively regulate FLI1 protein expression (Liu et al., 2019). The expression of miR145 increased after L1 treatment (Figure 4C). This data suggests that inhibition of FLI1 results in upregulation of miR145, further leading to downregulation of FLI1. Moreover, the GATA1, TFRC and GYPA genes are downstream targets of FLI1 to mediate erythroid differentiation, and their gene promotors are negatively regulated by Fli-1 (Athanasiou et al., 2000). Accordingly, the gene expression levels of GATA1, TFRC and GYPA were increased in L1-treated HEL cells (Figures 4D–F). In FLI1-knockdown cells (shFLI1) (Supplementary Figures 1A,B), the inhibition rate of the L1-treated group was greater than that of the scramble group (Figure 4I), and the IC_50_ values were 5.16 ± 1.23 for the shFLI1 group and 1.92 ± 0.25 for the scramble group. These data suggested that L1 mediated erythroid differentiation through the miR145/FLI1 pathway.

The PI3K/AKT signaling pathway is abnormally overexpressed in many cancers, including AML (Darici et al., 2020; He et al., 2021). As shown in several studies, FLI1 is a transcription factor involved in the activation of the AKT signaling pathway (Lakhanpal et al., 2010; Vecchiarelli-Federico et al., 2017). Western blotting analysis revealed that the expression of AKT and p-AKT was downregulated after L1 treatment (Figure 4G). RT‒qPCR revealed that the gene expression levels of CDKN1A and CDKN1B, which are downstream of the AKT pathway, were also increased by L1 (Figures 4H,I). These data suggested that L1 mediated cell growth through the FLI1/AKT pathway.

### 3.5 L1 induced cell apoptosis in dependence on HSP70

**FIGURE 5:**
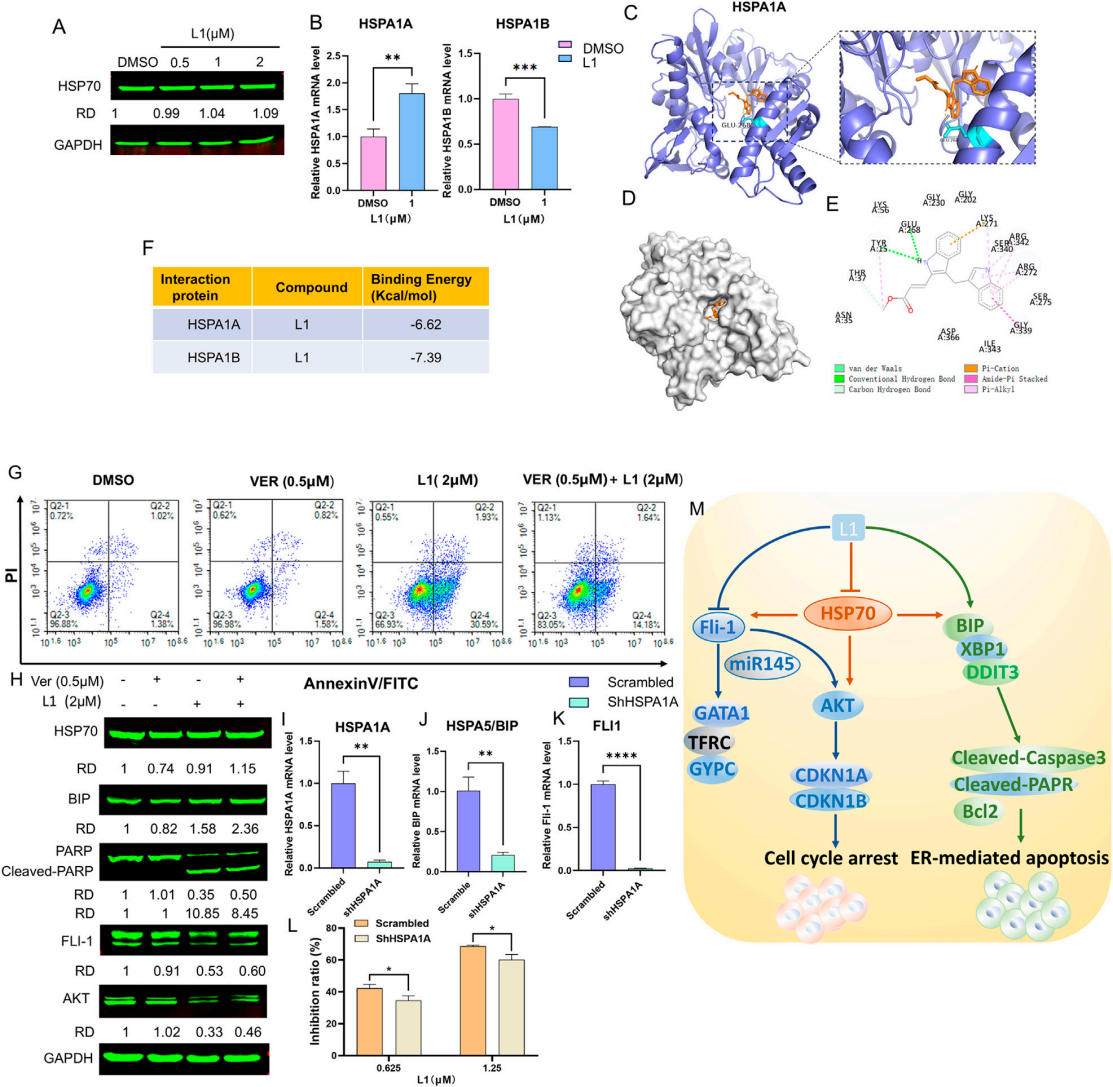
L1 induced cell apoptosis in dependence on Hsp70. **(A)** The expression level of HSP70 was detected by Western blot assay. The HEL cells were treated with vehicle control, L1 (0.5, 1, 2 µM) for 24 h. GAPDH expression was used as reference standard for quantification. The relative expression (RD) was used to quantified the protein expression. **(B)** The expression levels of HSPA1A and HSPA1B were detected by RT-qPCR. The HEL cells were treated with vehicle control (0.1%DMSO) or L1 (1 µM) for 24 h. **(C–E)** Docking analysis of the interaction between L1 and HSPA1A by AutoDock software. The hydrogen bond between L1 and amino acid residues Glu268. **(F)** The binding affinity and capacity for the active sites of HSPA1A and HSPA1B. **(G)** The cell apoptotic rate was detected by the flow cytometry assay after L1cotreated with HSP70 inhibitor VER155008 and L1. The HEL cells were treated with VER155008 (0.5 µM) and L1 (2 µM) for 72 h, and stained with annexin V-FITC and PI. **(H)** The protein expression levels of HSP70, BIP, PARP, Cleaved-PARP, FLI1 and AKT were detected by Western blot assay. GAPDH expression was used as reference standard for quantification. The relative expression (RD) was used to quantified the protein expression. **(I–K)** The expression levels of HSPA1A, BIP, FLI1 were detected by RT-qPCR. The HEL cells were treated with shHSPA1A knockdown. **(L)** The inhibition rates of L1 in the HEL cells with shHSPA1A knockdown were detected by MTT assay. The HEL cells were treated with vehicle control (0.1%DMSO) or L1 (0.625,1.25 µM) for 72 h *p < 0.05, **p < 0.01, ***p < 0.001, versus control, *n* = 3. **(M)** The anti-erythroleukemia mechanism of L1.

To explore the relationship between L1 and HSP70 (HSPA1A, HSPA1B), Western blotting and RT-q‒PCR were used to test the expression of HSP70 after L1 treated. The gene expression level of HSPA1A was increased and the gene expression level of HSPA1B was decreased, while protein expression level of HSP70 was almost unchanged (Figures 5A,B). The AutoDocking data revealed that L1 could bind to the HSPA1A protein around the ATP-binding domain (ABD), and the lowest binding energy was −6.62 kcal/mol (Figures 5C–F). The hydrogen bond was between L1 and the amino acid residue Glu268 of HSPA1A. L1 could also bind to the HSPA1B protein, and the lowest binding energy was −7.39 kcal/mol (Supplementary Figures 2A–D). The hydrogen bond was between L1 and the amino acid residue GLY339 of HSPA1B. The ATP-competitive HSP70 inhibitor VER155008 forms hydrogen bonds in the ATP binding pocket with the amino acid residues Arg272 and Arg342 (Schlecht et al., 2013). VER155008 did not induce cell apoptosis at the indicated concentration (VER155008, 0.5 µM) and was used to treat cells with L1 (2 µM), and the apoptosis rate of the cotreated group was decreased to 15.82% compared with that of the L1-treated group at 32.52% (Figure 5G). Western blotting analysis also revealed that the expression level of the apoptosis-related protein cleaved-PARP was decreased in the cotreated group (Figure 5H). The protein expression of HSP70 in the cotreated group recovered compared with that in the VER155008 alone group (Figure 5H). After HspA1A was knocked down in HEL cells) (Figure 5I), the cell inhibition rates were lower than those in the scramble groups when L1 was incubated with these knockdown cells for 72 h (Figure 5L). These data suggest that HSP70 may be involved in L1-induced apoptosis.

Interestingly, immunoblotting analysis revealed that the protein expression levels of AKT and FLI1 were higher in HEL cells co-incubated with L1 and the HSP70 inhibitor VER155008 than in L1-treated HEL cells alone (Figure 5H). These data suggested that L1 also regulated the FLI1/AKT pathway dependent of HSP70. Thus, L1 induced HSP70-dependent apoptosis and downregulated the expression of FLI1 to mediate its downstream signaling pathway (Figure 5M). We found that the gene expression levels of BIP and FLI1 were decreased in the HSPA1A-knockdown cells (Figures 5I–K). The mechanism by which HSP70 is associated with expression of BIP and FLI1 is an interesting area of research that may need to be further explored in future studies.

## 4 Discussion

Although chemotherapeutic medications are widely used for cancer treatment, serious side effects are harmful to patients. Natural chemicals derived from fruits, vegetables and spices are important sources for the development of novel anticancer medications with lower toxicity and greater efficiency. Recently, DIM, which has been derived from cruciferous vegetables such as broccoli, Brussels sprouts, and cauliflower, has been used as a cancer chemopreventive supplement to improve outcomes in clinical therapeutics (Reyes-Hernández et al., 2023). DIM has also shown great potential for anti-proliferative and anti-inflammatory activities in several types of cancers, involving NF-κB, Akt, Wnt, PI3K/Akt/mTOR, and AhR signaling (Biersack, 2020; Reyes-Hernández et al., 2023), and several synthetic derivatives of DIM with increased bioavailability have been used as active antitumor compounds (Biersack, 2020). For example, a DIM derivative DIM-C-pPhtBu, 1,1-bis(3′-indolyl)-1-(p-t-butylphenyl) methane, activates ER stress in many cancer cells, including human oral cancer (Shin et al., 2011), pancreatic cancer (Abdelrahim et al., 2006), colon cancer (Chintharlapalli et al., 2004), ovarian cancer (Lei et al., 2006) and breast cancer (Vanderlaag et al., 2008; Vanderlaag et al., 2010). Mechanistically, DIM-C-pPhtBu was shown to inhibit growth and induce apoptosis in cancer cells through both PPAR gamma-dependent and PPAR gamma-independent pathways (Chintharlapalli et al., 2006; Lei et al., 2006). Recently some researchers have found DIM-C-pPhtBu induced lysosomal dysfunction, leading excessive mitophagy in head and neck cancer HNC cells (Kang et al., 2021). Few studies have explored the effects of DIM and its derivatives on leukemia. Only several researchers have shown that DIM is a potential compound for therapeutic application in human T-ALL cells (Shorey et al., 2012) and that a ring-substituted diindolylmethane derivative (DIM #34), 1,1-bis [3'-(5-methoxyindolyl)]-1-(p-t-butylphenyl) methane, selectively induced apoptosis in AML cells through regulation of the extracellular signal-regulated kinase and the PPAR gamma-dependent signaling pathways (Contractor et al., 2005). In our study, a new DIM derivative L1, methyl (E)-3-(3-((1H-indol-3-yl)methyl)-1H-indol-2-yl)acrylate, was synthesized in our group. L1 showed very low IC_50_ value of 1.15 ± 0.03 µM in the erythroleukemia HEL cells. FLI1 was a specific target in the erythroleukemia HEL cells (Ben-David et al., 1990), and FLI1 inhibitors were developed to treat erythroleukemia in our previous study (Li et al., 2015) Herein, we showed that L1 selectivity and specificity inhibited the erythroleukemia HEL cell line rather than in breast cancer cell lines. L1 exhibited significant antileukemic activities through modulation of the FLI1/AKT pathway, which displayed a novel and different mechanism from other DIM derivatives.

The unfolded protein response (UPR) leads to the accumulation of misfolded/unfolded proteins toward a degradative pathway to rebuild ER homeostasis, while persistent ER stress triggers pro-apoptotic conditions (Gong et al., 2017). ER stress modulates tumor progression and plays an important role in tumor development by initiating a persistent UPR as an adaptive pathway (Zhang et al., 2024). Previous reports demonstrated that modulating ER pressure sensors or UPR-related factors obviously improved the sensitivity of malignant tumors to cytotoxic agents, targeted drugs and immunotherapy (Zhang et al., 2023). Therefore, focusing on ER stress-related pathways is highly important for anticancer therapy. Some studies have shown that DIM inhibits cancer cells through inducing ER stress (Sun et al., 2004; Savino et al., 2006; Zhang et al., 2017; Heo et al., 2018; Munakarmi et al., 2021). DIM inhibited hepatocellular carcinoma by activating caspase-dependent apoptosis via ER stress and the UPR (Munakarmi et al., 2021). DIM modulates cyclin D1 expression by activating ER stress in colorectal cancer cells (Zhang et al., 2017). DIM-C-pPhtBu increases ER stress by inducing the protein expression of BIP and CHOP in many cancers (Abdelrahim et al., 2006; Shin et al., 2011; Kang et al., 2021). Targeting ER stress is a potential therapeutic strategy for AML treatment. The (epi)genetic modifications and genomic instability, oncogenic signaling, and metabolic rewiring in leukemia are involved in the activation of ER stress and each of the three UPR signaling pathways (IRE1α, PERK, and ATF6α) (Féral et al., 2021). High expression levels of XBP1 and BIP have been detected in certain AML subtypes (Schardt et al., 2011), while DDIT3(CHOP) is a specific protein involved in ER stress. Transcription factors such as ATF4, ATF6, and XBP1 are translocated into the nucleus to increase DDIT3 transcription. This process leads to the initiation of the apoptotic signaling pathway via the activation of the expression of the apoptosis-related protein cleaved-PARP and the suppression of the antiapoptotic protein B-cell lymphoma-2 (Bcl-2) (Zhang et al., 2023). In our study, we found that the DIM derivative L1 induced ER stress-mediated apoptosis in erythroleukemic HEL cells, with upregulated RNA expression of XBP1 and DDIT3 and upregulated protein expression of BIP. Meanwhile, the expression of the apoptosis-related protein cleaved-Casepase3 and cleaved-PARP were activated and Bcl-2 was decreased in the L1-treated HEL cells.

GRP78/BIP, a member of the heat shock protein 70 (HSP70) family, is located at the membrane of the endoplasmic reticulum. GRP78 acts as an important sensor in the activation of the UPR and interacts with the UPR proteins IRE1 and PERK, preventing the binding of GRP78 to its cochaperones (Kopp et al., 2019). The GRP78 protein shares 60% homology with the proteins of the HSP70 family, including the ATP-binding domain (ABD) and substrate-binding domain (SBD) (Ibrahim et al., 2019). In this study, we showed that L1 induced ER stress and upregulated the expression of GRP78. HSP70/HSPA1 is encoded by the stress-inducible genes (HSPA1A,HSPA1B and HSPA1L) (Ucisik-Akkaya et al., 2010). The coding regions of HSPA1A and HSPAIB are closely linked, stress-inducible and intronless genes, only promoter and 3′UTR sequences are different. According to their sequences, HSPA1A and HSPAIB have more than 99% identical sequences except two (E110D, N499 S) of their 641 amino acids (Daugaard et al., 2007; Smith et al., 2007). HSPA1A (HSP70-1) is expressed at low levels in unstressed normal cells and overexpressed at the plasma membrane in several types of tumor cells to promote tumor development (Vostakolaei et al., 2021). HSPA1B was induced in the in erythroleukemia cells and knockdown of HSPA1B accelerated leukemic cell proliferation (Wang et al., 2024). In this study, the gene expression level of HSPA1A was increased and the gene expression level of HSPA1B was decreased after L1 treated. Interestingly, the HSP70 inhibitor VER155008 at the designated concentration (0.5 µM) inhibited the expression of the HSP70 protein but did not directly induce apoptosis, reversing the increase in the percentage of apoptotic cells in the L1 and VER155008 cotreated group. The HSP70 inhibitor VER155008 targets the binding pocket and forms hydrogen bonds with the amino acid residues Arg272/Arg342 of HSP70 (Schlecht et al., 2013). In the AutoDocking analysis, L1 was shown to be inserted into the ATP binding pocket of HSPA1A and HSPA1B, which formed the hydrogen bond near the binding domain of VER155008 and may work as an antagonist of VER155008. Although the protein expression level of HSP70 was stable after L1 treatment, the inhibition rates were decreased in HSPA1A-knockdown HEL cells. Therefore, these data suggest that HSP70 may be associated with the ER stress-induced apoptosis induced by L1. However, the precise mechanism of L1 how to bind HSP70 (HSPA1A and HSPA1B) and then participate in ER stress-induced apoptosis needs to be further explored in future studies.

FLI1, a transcription factor (TF), is involved in the development of the hematopoietic system, and its overexpression leads to the progression of erythroleukemia. FLI1 contributes to the regulation of proliferation, differentiation and inflammation (Athanasiou et al., 2000; Li et al., 2015). Therefore, FLI1 is considered a specific and important target for erythroleukemia treatment. In our study, we found that L1 inhibited the protein expression of FLI1 and regulated its downstream gene expression of GATA1, TFRC and GYPA, which are associated with erythroid differentiation. In previous research, our group showed that downregulation of FLI1 protein expression by FLI1 inhibitors may be involved upregulation of miR145 through a posttranscriptional mechanism (Liu et al., 2019), and miR-145 is a negative regulator that mediates the expression of FLI1. Herein, we revealed that the downregulated expression of FLI1 was accompanied by increased expression of miR145 with L1 treatment. Moreover, FLI1 is overexpressed in the erythroleukemia cell lines, which leads to increased cell proliferation partly through the activity of the AKT pathway (Vecchiarelli-Federico et al., 2017). We also revealed that the protein expression levels of AKT were decreased and that the RNA expression levels of the downstream genes CDKN1A and CDKN1B were increased in the L1-treated group. Accordingly, these data demonstrated that L1 inhibited HEL cell growth in part through the miR145/FLI1/AKT signaling pathway.

## 5 Conclusion

DIM is a cancer chemopreventive supplement that can improve outcomes in clinical therapeutics**.** In this study, we revealed the anti-erythroleukemia activities of a novel DIM derivative L1 *in vitro*. L1 induced ER stress-mediated apoptosis and mediated the FLI1/AKT pathway in HEL cells. L1 was then a potential candidate for erythroleukemia therapies. However, further improve in the bioavailability of L1 and its effectiveness *in vivo* may be needed in future studies to demonstrate its therapeutic benefit.

## Data Availability

The original contributions presented in the study are publicly available: GEO repository, accession number GSE303419 (https://www.ncbi.nlm.nih.gov/geo/query/acc.cgi?acc=GSE303419).

## Supplementary material

SUPPLEMENTARY FIGURE S1
**(A)** The RNA expression level of FLI1 were detected by RT-qPCR. **(B)** The protein expression level of FLI1 was detected by Western blot assay. GAPDH expression was used as reference standard for quantification. The relative expression (RD) was used to quantified the protein expression. **p* < 0.05, ***p* < 0.01, ****p* < 0.001, versus control, *n* = 3.

SUPPLEMENTARY FIGURE S2
**(A–D)** Docking analysis of the interaction between L1 and HSPA1B by AutoDock software. The hydrogen bond between L1 and amino acid residues Gly339. The binding affinity and capacity for the active sites of HSPA1B.
