# Supplementary figures and images for: L1, a 3,3′-diindolylmethane-derivative, induced ER stress-mediated apoptosis and suppressed growth through the FLI1/AKT pathway in erythroleukemia HEL cells
Source: Front Pharmacol. 2025 Aug 1;16:1564199. doi: 10.3389/fphar.2025.1564199 (PMC12354999; doi:10.3389/fphar.2025.1564199)

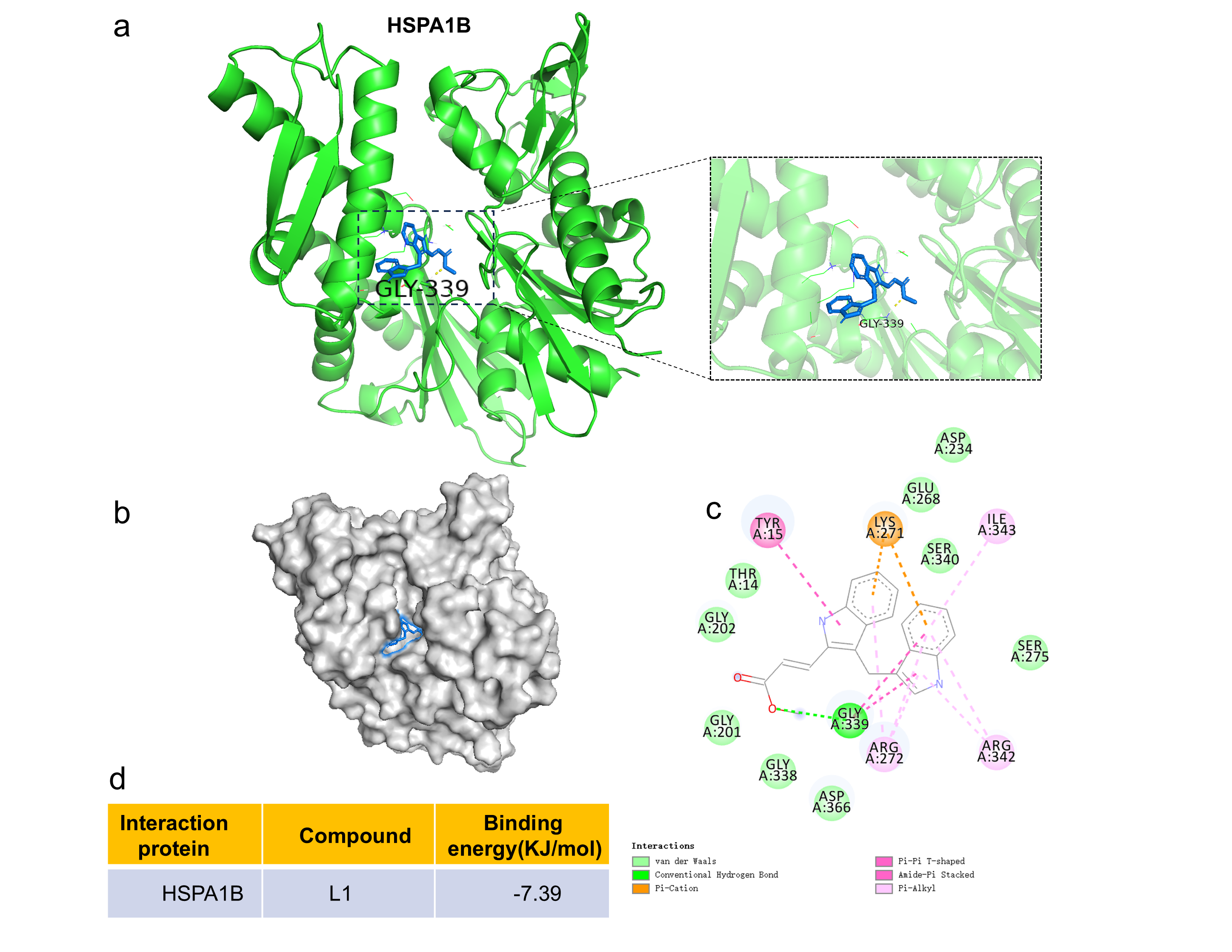

Supplement: Supplementary file 1 [file Image2.tif]

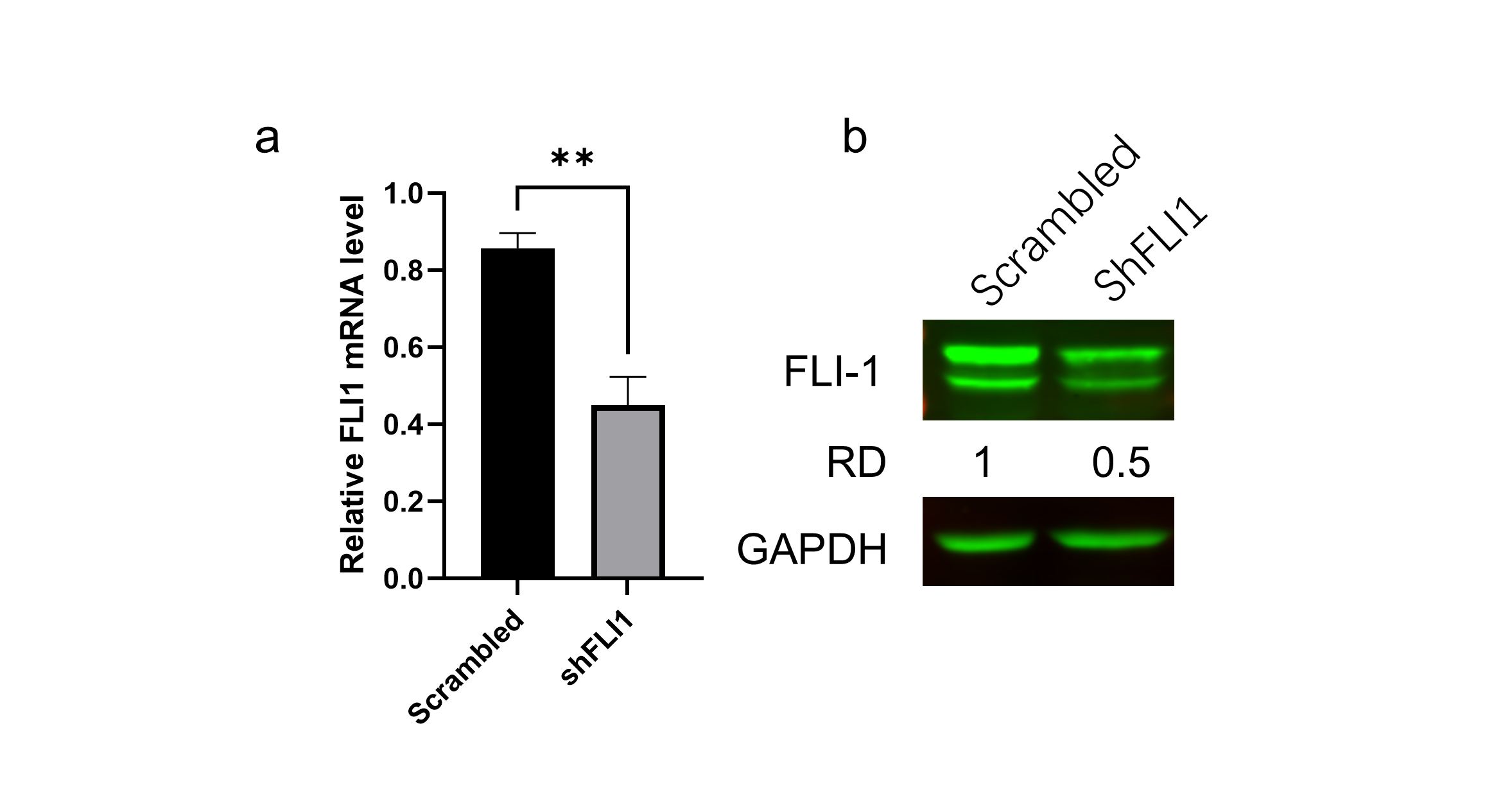

Supplement: Supplementary file 2 [file Image1.tif]
